# Eutopic echoes ectopic: organoid-based evidence of shared hormone dysregulation in endometriosis

**DOI:** 10.1186/s12958-026-01559-4

**Published:** 2026-04-23

**Authors:** Juste Cioraityte, Lennard Schröder, Zeinesh Beimenbetova, Anna T. Rückl, Susanne Beyer, Johanna Becker, Thomas Kolben, Sven Mahner, Mirjana Kessler, Simon Keckstein

**Affiliations:** Department of Obstetrics and Gynecology, University Hospital, Ludwig-Maximilians-University Munich, Marchionini Str. 15, Munich, 81377 Germany

**Keywords:** Endometriosis, Organoid, Steroid hormone, WNT signaling, Estrogen receptor alpha, Estrogen receptor beta, Progesterone receptor, Aromatase

## Abstract

**Background:**

Endometriosis is a highly prevalent, hormone-driven chronic disease characterized by the growth of endometrial tissue outside the uterine cavity. Despite its impact, the mechanisms underlying the disease’s pathogenesis remain poorly understood. We hypothesized that not only the ectopic endometrium of women with endometriosis differs from that of women without the disease, but also the eutopic endometrium. Accordingly, this study aimed to identify potential differences in basal expression of hormone receptors, their responses to hormonal treatment, as well as general morphology and growth factor dependencies among organoids derived from the endometrium of women without endometriosis and from the eutopic and ectopic endometrial tissues of patients with endometriosis.

**Methods:**

Patient-derived organoids were established from the endometrium of women without endometriosis and from the eutopic and ectopic endometrial tissue of patients with endometriosis. To determine optimal culture conditions, primary isolates were seeded in parallel in medium containing the WNT signaling activators WNT3a and R-spondin-1 (RSPO1) or in medium supplemented with RSPO1 alone to evaluate WNT pathway requirements. Organoids were exposed to 17β-estradiol (E2), progesterone (P4) with cAMP, and the WNT inhibitor XAV939. The expression of estrogen receptor alpha and beta (*ESR1*,* ESR2*), progesterone receptor (*PGR*), and aromatase (*CYP19A1*) was measured via qPCR. Organoid morphology, growth, and aromatase expression were determined by brightfield and immunofluorescence imaging. Relative gene expression was calculated using the comparative CT method (ΔΔCT) to evaluate hormone responses across groups.

**Results:**

Organoids derived from control donors and from eutopic endometrium of endometriosis patients exhibited robust long-term growth in medium supplemented with WNT3a and RSPO1, whereas ectopic endometriotic organoids proliferated best in medium containing only RSPO1, indicating reduced dependence on exogenous WNT pathway activation.

Basal *ESR1* and *PGR* expression was lower in lines from endometriosis patients than in control lines, indicating reduced E2 sensitivity and P4 responsiveness. *ESR2* levels were uniformly low across groups. Upon E2 stimulation, *ESR1* expression was consistently suppressed, whereas *PGR* expression was strongly induced in ectopic organoids (29.9-fold, ***p < 0.001), in eutopic organoids (13.5-fold, *p = 0.016), and to a lesser extent in organoids from women without endometriosis (5.2-fold, *p* = 0.069). P4-mediated downregulation of *PGR* required WNT inhibition. The effectiveness of amended hormonal stimulation with XAV939 in downregulating *PGR* in organoids was validated at the protein level via Western blotting (WB). Sporadic aromatase expression was detected exclusively in organoids derived from endometriosis patients.

**Conclusions:**

These findings demonstrate that the eutopic endometrium of endometriosis patients exhibits altered responses to hormone stimulation. In this regard, it mirrors the characteristics of ectopic lesions more closely than those of endometrium from women without endometriosis. These results suggest that the endometrium plays an intrinsic role in the pathophysiology of endometriosis, providing a strong basis for future mechanistic studies.

**Supplementary Information:**

The online version contains supplementary material available at 10.1186/s12958-026-01559-4.

## Background

Endometriosis is one of the most common benign gynecological diseases in women of reproductive age, characterized by the presence of endometrial tissue outside the uterine cavity. Despite its prevalence of ∼10%, many aspects of its pathogenesis remain elusive [1]. Like healthy endometrium, ectopic tissue is modulated by hormones, mainly through the trophic effects of estrogen dominating the cycle’s proliferative phase and the differentiation-regulating properties of progesterone in the secretory phase. These effects are primarily mediated by estrogen receptor alpha (*ESR1*), beta (*ESR2*), and progesterone receptor (*PGR*). Here, we assessed the basal and hormonally modulated expression of these receptors in patient-derived epithelial organoids generated from endometrium of women without endometriosis (EMT), as well as from eutopic (euEMT) and ectopic (ecEMT) endometrial tissue of endometriosis patients.

*ESR1* mediates the effects of estrogen, primarily of 17β-estradiol (E2), in the endometrium, driving epithelial proliferation and the induction of *PGR* synthesis, which are necessary for the subsequent secretory phase [2–4]. Reports in the literature indicate that *ESR1* expression is reduced in endometriotic tissues, resulting in impaired estrogen signaling; however, the data are mainly from histological studies focused on the stromal compartment [5]. 

*ESR2* has been shown to modulate *ESR1* activity, exerting antiproliferative effects that counterbalance the effects of *ESR1* [6, 7]. There is strong evidence that the *ESR1*-to-*ESR2* ratio is > 1 in healthy endometrium, but the opposite in endometriosis [8–12]. Accordingly, the presumed overexpression of *ESR2* inhibits apoptosis by interacting with the cellular apoptotic machinery and inflammasome. As demonstrated in a mouse model, this may support the ability of endometriotic cells to evade immune surveillance, facilitating ectopic lesions [13]. However, prominent interspecies differences, such as the mouse’s 4–5 day estrus cycle and the absence of endometrial shedding, limit the strength of direct analogies to a human disease [14–17]. 

The *PGR* balances differentiation, which is necessary for a receptive endometrium [18]. It is acknowledged that progesterone resistance plays a pivotal role in endometriosis pathophysiology, leading to persistence and progression of endometriotic lesions with its exact mechanism remaining poorly understood [5, 19]. Progesterone (P4) and its analogues are commonly used for symptomatic treatment by suppressing ovulation and limiting estrogen-driven stimulation of ectopic tissue. Therefore, progesterone resistance poses an additional challenge in this context [20, 21]. 

Dysregulation of these receptors has been implicated in the development, continuance, and progression of endometriosis, contributing to impaired hormonal responsiveness and altered tissue behavior [5, 11]. Given the essential role of hormonal stimulation, we also assessed the possible contribution of local estrogen biosynthesis. Aromatase, encoded by the *CYP19A1* gene, catalyzes the conversion of androgens to estrogens. While virtually absent in healthy endometrium, aberrant aromatase expression has long been suggested as a driver of endometriosis pathogenesis [22, 23]. 

Recent advancements in patient-derived organoids have enabled 3D models that successfully recapitulate the cellular architecture, functional responses, and intercellular communication. While endometrial organoids have been described from donors with and without endometriosis, prior studies have not examined hormone receptor expression in response to hormonal treatment [24–29]. Previous publications have demonstrated the importance of targeted WNT/β-catenin (WNT) pathway activation for the successful establishment of organoids. There, low WNT stimulation enhanced the differentiation capabilities of endometrial organoids, whereas high activation of the pathway promoted stemness [24, 30]. In the present study, the cultivation requirements across organoid groups were compared.

In our established epithelial organoids from women without endometriosis, as well as eutopic and ectopic endometrium from women with endometriosis, we focused on investigating *ESR1*, *ESR2*, and *PGR* expression profiles, as well as evaluating aromatase presence. A direct comparison revealed distinct molecular profiles and signaling requirements between organoids from endometrium of women without endometriosis and endometriosis-derived organoids, thereby supporting our hypothesis that euEMT differs from EMT in both molecular and functional features. Notably, euEMT exhibited characteristics that not only differed from those of EMT but also more closely resembled those of ecEMT. This observation might support the concept of an autonomous epithelial defect contributing to the pathophysiology of endometriosis.

## Methods

This experimental laboratory study aimed to investigate potential differences in hormone receptor expression and hormone responsiveness in patient-derived epithelial organoids established from women with and without endometriosis.

### Procurement of human material

Human endometrial and endometriotic samples were obtained from the Department of Gynecology and Obstetrics, LMU University Hospital Munich, Germany. The scientific use of these samples for research was approved by the Ethics Commission of LMU Munich (17–0471). Patients with suspected endometriosis or infertility who underwent hysteroscopy and laparoscopy at our center for the evaluation of these conditions were informed about the study preoperatively and consented to the donation of tissue for research and biobanking. Fragments were obtained from laparoscopic surgery as part of the diagnostic endometriosis assessment; further, a portion of the endometrial curettage - usually performed to exclude chronic endometritis - was allocated for study purposes. Only patients with inconspicuous findings in laparoscopy, hysteroscopy, and in the histology of the endometrial curettage were included in the group of women without endometriosis. The cells were isolated from samples within 24 h of removal.

### Tissue processing and organoid culture

Organoids were generated and cultured as described in Kessler et al. [31]. The samples were washed with Dulbecco’s phosphate-buffered saline (DPBS, Gibco) (Supplement 1), and small pieces were separated for paraffin embedding. Samples with recognizable endometrium were left intact, otherwise chopped, and incubated with 0.5 mg/ml Collagenase I (Gibco) and 3 mM ROCK inhibitor (Bio-techne) (1 h, 37 °C). If present, discernible endometrium was scratched off with a scalpel, and excessive connective tissue was removed. The cell solution was transferred to a 5 ml tube (Biozym) and mixed 1:1 with ADF++, containing Advanced DMEM/F12 Reduced Serum Medium (Gibco) + 1% GlutaMAX (Thermo Scientific) + 1% HEPES 1 M (Gibco) + 1% Penicillin/Streptomycin (Invitrogen). The suspension was centrifuged (5 min, 350 x g), and cells were separated by filtering through a 400 μm cell strainer (PluriSelect). Persistent erythrocytes were depleted with 5 mL Red Blood Cell Lysis Buffer (Roche). After pelleting, cells were resuspended in Cultrex Reduced Growth Factor Basement Membrane Extract, Type 2 (R&D Systems) and seeded in prewarmed 24 (48)-well plates (Falcon) with ~ 60,000 (~ 30,000) cells/well in a 50 µl (25 µl) drop. The plate was placed in a humidified incubator (30 min, 37 °C, 5% CO_2_) to polymerize Cultrex. After the addition of the appropriate medium (Supplement 2), cells were returned to the humidified incubator. The medium was changed every 3–4 days. Organoids were passaged and expanded approximately every 14 days at a 1:2–1:5 ratio. Release from Cultrex was accomplished by removing the culture medium, adding ice-cold ADF + + to the wells, and mechanically scratching with a 1000 µl pipette tip (Sapphire). After pelleting, cells were washed until Cultrex remnants were removed. Subsequently, organoids were resuspended in 300 µl/well TrypLE™ Express Enzyme (Gibco) and incubated (10–15 min, 37 °C), during which organoids were vortexed every 2 min for 10 s. The enzyme was neutralized with cold ADF++ (1:1), cells pelleted, and seeded in Cultrex as stated before.

### Organoid stocks

At ~ 7 days of age, organoids from 2 to 3 wells of a 24-well plate were dissociated from Cultrex as described above. After centrifugation, the pellet was resuspended in 500 µl/well Cryo-SFM Plus freezing medium (PromoCell). The cryotube (Thermo Scientific) was placed in a freezing container (Mr. Frosty, ThermoFisher) (> 24 h, -80 °C) before being transferred into liquid nitrogen.

### Immunofluorescence staining

Parts of the native tissues used for organoid establishment were fixed for ≥ 24 h in 4% PFA for comparative analysis. Fixed organoids and tissue were subjected to paraffin-embedding, sectioned at 2 μm and 6 μm, respectively, and mounted onto microscope slides (Superfrost Plus Adhesion, Epredia). The samples were deparaffinized using descending alcohol concentrations. Afterwards, the slides were washed in DPBS on a shaker (3 × 5 min), likewise after every one of the following steps. Antigen retrieval was performed in a cuvette with TRIS (Roth) – EDTA (Sigma) buffer (pH 9.0, 30 min) in a steamer (Vitacuisine compact, Tefal). Permeabilization was achieved with Triton X-100 (Sigma-Aldrich) (15 min) and blocking with Blocking Solution 10% (Normal Donkey Serum, Abcam) in Antibody Dilution Reagent (DAKO) (30 min). Finally, the primary antibodies were applied and incubated in a humid chamber (4 °C, 12 h). The secondary antibodies were administered and incubated in the dark (2 h, RT), followed by a nucleic acid dye DAPI (1:1000, Thermo Scientific) (5 min) staining. After the last washing step, coverslips were mounted on slides with Mowiol 4–88 (Roth), left to dry (24 h, RT), and sealed with nail polish. Immunofluorescence images were captured via the Keyence BZ-X810 fluorescence microscope.

### Stimulation experiments

We selected a 13-day period for assessment of our experiments, as this provides a stable and reproducible differentiation state for evaluating hormonal responses in organoids [31]. For hormone stimulation assays, organoids were released from Cultrex and disrupted into single cells enzymatically (TrypLE™, 15 min, 37 °C) and mechanically by vortexing and passing through a G26 cannula (0.45 × 25 mm, Sterican). The cells were counted and seeded in a 24-well plate at 50,000 cells / 50 µl Cultrex. On day 4, the organoids were treated with 10 nM 17β-estradiol (E2, Sigma-Aldrich), and on day 6 either exposed to 10 nM E2, additionally combined with 1 µM progesterone (P4, Sigma-Aldrich) and 1 µM 8-bromadenosine-3`5`-cyclic monophosphate (cAMP, Biolog), further complemented with 10 µM XAV939 (Sigma-Aldrich) or left untreated as control (Fig. 3A). The organoid culture medium (Supplement 2), augmented with the respective compounds, was changed on day 9. On day 13, the organoids were harvested for RNA isolation.

Data were generated from 5, 4, and 6 independent biological experiments for EMT, euEMT, and ecEMT, respectively. Organoid lines were derived from 9 tissue samples obtained from 7 individual donors (Supplement 3). To ensure reproducibility, different passages of organoids originating from the same donor were stimulated across experiments, consistently yielding comparable results. For each sample and condition, organoids were cultured in duplicate wells and pooled before RNA extraction. qPCR was performed in technical triplicate, and averaged CT values were used for analysis. Brightfield images were captured using a Leica DMi1 microscope.

### RNA isolation

After the organoids were retrieved from Cultrex as previously described, the organoid pellet was resuspended in a 1:100 β-mercaptoethanol (Sigma-Aldrich)/RLT-buffer (Qiagen) suspension, and the lysate was passed 3–5 times through a G26 cannula.

Organoid RNA was isolated via the RNeasy Mini Kit (Qiagen), following the manufacturer’s protocol. RNA concentration was measured using the Qubit 4 Fluorometer and Qubit RNA Broad Range Assay (Qiagen).

### RT-qPCR

Of the obtained RNA, complementary DNA (cDNA) was transcribed using a cDNA Synthesis Kit (Biozym) and diluted to 5 ng/µl. The real-time quantitative polymerase chain reaction (RT-qPCR) was performed in triplicate per primer/sample. For this, we used gene-specific TaqMan primer assays (Thermofisher) diluted in TaqMan Fast Universal PCR Master Mix (Thermofisher). Each reaction enclosed 7.5 µl Master Mix, including the probe and 2.5 µl cDNA (5 ng/µl). Amplification was performed using the 7500-Fast Real-Time PCR System (Applied Biosystems) and analyzed with the 7500 Fast System Software (Applied Biosystems, v1.5.1).

### Protein extraction and western blot

After the organoids were retrieved from Cultrex as previously described, the organoid pellet was resuspended in a 5% β-mercaptoethanol (Sigma-Aldrich) / 2x Laemmli buffer (Biorad), the lysate was passed 3–5 times through a G26 cannula and boiled in a preheated thermomixer (10 min, 96 °C, 350 rpm).

For protein separation, samples were resolved by SDS–PAGE using 10% polyacrylamide gels. A prestained molecular weight marker (PageRuler Plus, Thermofisher) and 12 µl protein per lane were loaded onto the gels. Electrophoresis was performed in Tris–glycine–SDS running buffer. Proteins were transferred to the membrane (0.2 μm PVDF) using the semi-dry Trans-Blot turbo system (Biorad). Afterwards, blocking was performed with TBS-Tween 0,05% / 1% BSA / 3% milk powder (1 h, RT). The primary antibodies were diluted in the blocking solution and incubated on the shaker overnight (4 °C). After washing with TBS-Tween 0,05%, the membrane was incubated with the respective secondary HRP-conjugated antibody (1 h, RT), followed by washing with TBS-Tween 0,05%. Proteins were detected by use of the Cytiva Amersham ECL Prime Western-Blot-Detection Reagent (Thermo Scientific) on the ChemiDoc MP Imaging System (Biorad).

### Statistical analysis

The datasets supporting the conclusions of this article are included within the article and its additional files. Relative gene expression, measured via RT-qPCR, was calculated using the comparative CT method (ΔΔCT) (Supplement 4). Target gene expression levels were normalized to the glyceraldehyde-3-phosphate dehydrogenase (*GAPDH*) expression in each organoid triplicate sample. ΔΔCT was estimated as the mean ΔCT of independent biological replicates subtracted from the mean ΔCT of untreated organoids from patients without endometriosis (EMT). Fold change was then determined as 2^−(ΔΔCT)^. Standard deviation (SD) of the triplicate data for the target genes and *GAPDH* was calculated as

$$SD=\sqrt{\frac{\sum\:{\Delta\:}\mathrm{C}\mathrm{T}-\overline{{\Delta\:}\mathrm{C}\mathrm{T}}}{n-1}}\;and\;{SD}_{{\Delta\:}{\Delta\:}\mathrm{C}\mathrm{T}}=\:\sqrt{{\left({SD}_{GAPDH}\right)}^{2}+{\left({SD}_{target\:gene}\right)}^{2}}$$

To account for high donor-dependent variability and reduce the impact of extreme expression values, intergroup fold changes were compared using geometric means and are displayed in figures accordingly. Normal distribution of the data was preliminarily investigated using the Shapiro-Wilk test. Statistical analysis was performed on log-transformed values. For normally distributed data and the comparison of two groups, paired t-tests were utilized, whereas for groups of three with one influencing factor, a Welch’s analysis of variance (ANOVA) was performed. For two influencing factors, a two-way ANOVA was completed. Suppose the Shapiro-Wilk test was negative in any of the analyzed groups, either a Kruskal-Wallis test or a mixed-effects analysis was undertaken, depending on group size. All calculations were performed using GraphPad Prism (v10.3.1) and Microsoft Excel (v16.78). Statistical significance was defined as *p* < 0.05.

## Results

**Fig. 1:**
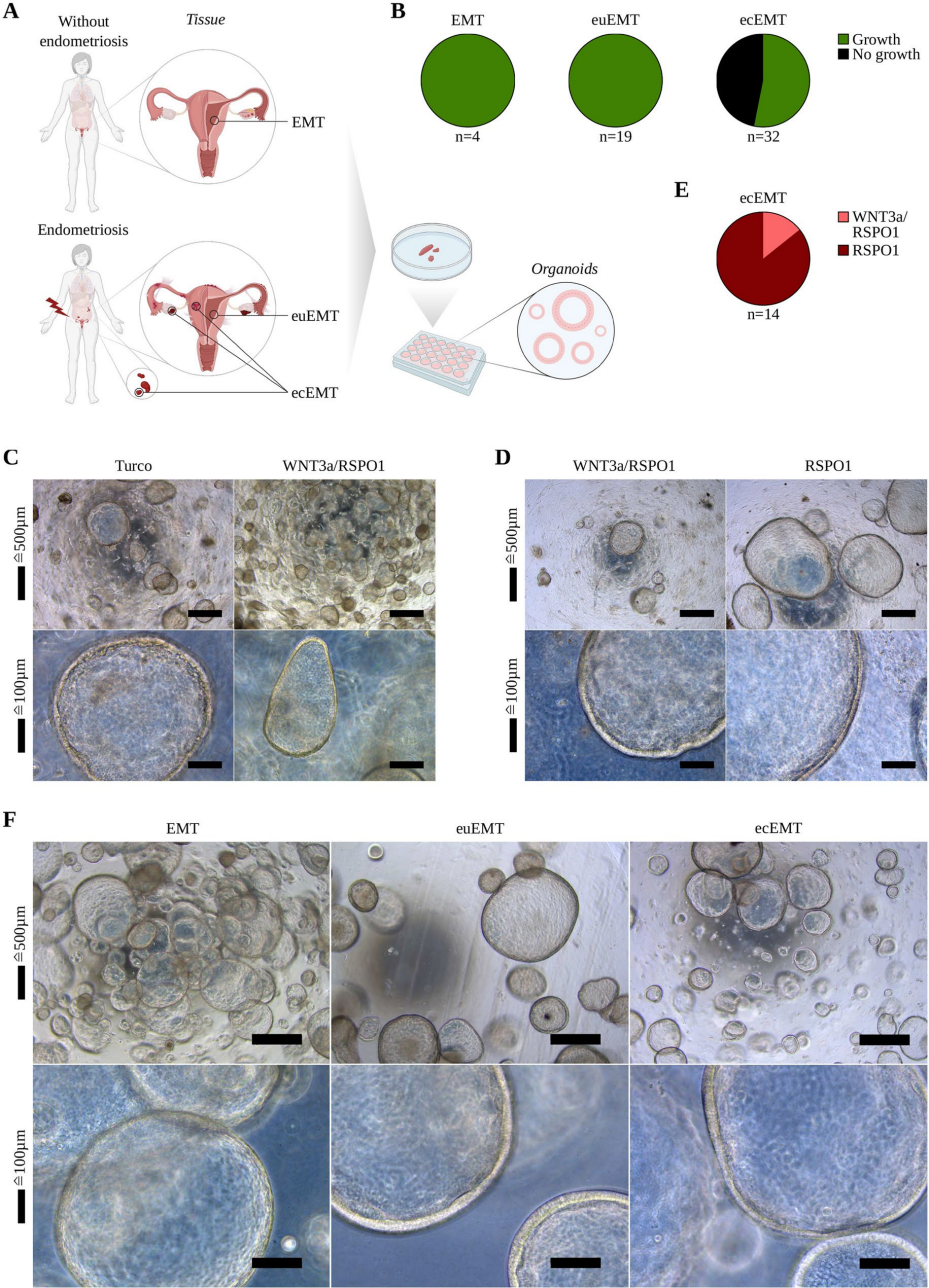
Establishment of patient-derived organoids and comparison of different WNT agonist containing media on organoid growth. **A** Schematic overview of tissue origin. Biopsies from EMT, as well as from euEMT and ecEMT tissue of endometriosis patients, were used to establish organoids. **B** Summary of success rates in organoid formation by originating tissue type. Organoids were successfully generated from 100% of EMT (*n* = 4), 100% of euEMT (*n* = 19), and 53.1% of ecEMT (*n* = 17/32) biopsies. **C**, **D** Representative brightfield images showing organoid morphology in different media. Scale bars as indicated. **C**, euEMT. **D**, ecEMT. **E** Effects of different WNT agonist supplemented media on ecEMT organoid growth. A superiority of the RSPO1-only medium was observed in 86% of ecEMT samples (*n* = 12/14). **F** Representative brightfield images comparing EMT, euEMT, and ecEMT organoids at passage 3, day 13. In this case, euEMT and ecEMT organoids are derived from the same donor. Compared to EMT, both euEMT and ecEMT organoids appear reduced in size and number, with ecEMT organoids being more numerous but smaller than those from euEMT. Scale bars as indicated. EMT = endometrium from women without endometriosis; euEMT = eutopic endometrium from endometriosis patients; ecEMT = ectopic endometrium from endometriosis patients

Forty patient-derived organoid lines were generated from 55 biopsies. Three subgroups were defined: 4/4 from endometrium of women without endometriosis (EMT) and 36/51 from endometriosis patients – 19/19 from eutopic endometrium in-utero (euEMT) and 17/32 from ectopic lesions (ecEMT), including endometriotic cysts, peritoneal endometriotic lesions, and deep infiltrating endometriosis (Fig. 1A, B).

### Modulations of a WNT activating environment on proliferative organoid potential

Dependency on active supplementation of a WNT agonist for organoid growth has been widely reported for different organ systems [31–35]. Slightly varying protocols have been published for endometrial organoids from women without endometriosis and fallopian tube organoids by Turco et al. [25] and Kessler et al. [31]. In this study, a parallel experimental testing of two formulations was performed immediately after progenitor isolation from endometrial tissue: one medium contained the canonical WNT ligand WNT3a [36] combined with R-Spondin 1 (RSPO1) [37], a potentiator of WNT/β-catenin signaling via LGR receptors. The other medium included RSPO1 alone, thereby providing comparatively lower exogenous activation of the WNT pathway. The WNT3a/RSPO1 medium promoted greater organoid formation efficiency, compared to the formulation containing RSPO1 only (Fig. 1C). As the growth advantage was preserved long-term after passaging, the WNT3a/RSPO1 medium was initially chosen as the optimal supplement for all isolation protocols (Fig. 1D).

However, parallel processing of eutopic and ectopic tissue from endometriosis patients revealed reduced organoid formation and growth arrest during passaging in the latter case. Consequently, we adapted the experimental design to reduce the WNT pathway-promoting signal in the medium. In direct media comparison, 86% (12/14) of the ecEMT organoids exhibited superior growth in the RSPO1 medium (Fig. 1E). These findings indicate that EMT and euEMT organoids require higher exogenous supplementation of WNT pathway agonists, whereas ecEMT organoids benefit from comparatively lower WNT promoting conditions in RSPO1-only medium. Furthermore, brightfield imaging at unified time points revealed morphological differences across groups: euEMT and ecEMT organoids were smaller and less abundant than EMT organoids, with ecEMT organoids appearing more numerous, yet consistently smaller than euEMT organoids (Fig. 1F).

**Fig. 2:**
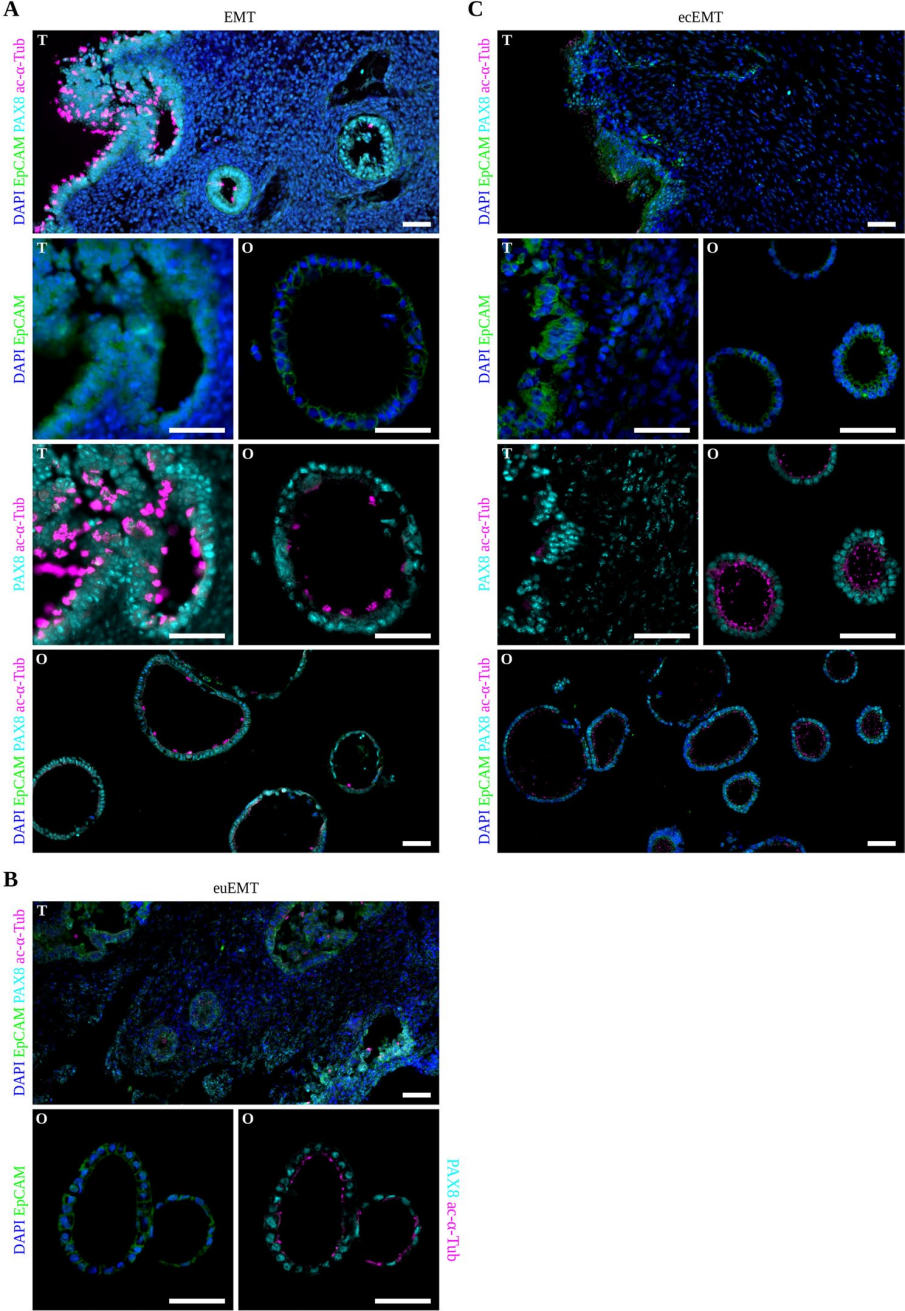
Immunofluorescence validation of epithelial identity and resemblance of organoids to corresponding tissue. Representative immunofluorescence images of native tissue (T) and organoids (O) from (**A**) EMT, (**B**) euEMT, and (**C**) ecEMT (here: peritoneal endometriotic lesion). DAPI (blue) marks DNA. EpCAM (green) confirms epithelial identity. Tissue epithelium and organoids contain both secretory cells positive for PAX8 (cyan) and ciliated cells positive for acetylated alpha-tubulin (ac-α-Tub, magenta), thereby confirming Muellerian lineage and differentiation capability. In native tissues, EMT and euEMT exhibit organised glandular structures, whereas ecEMT lacks glandular architecture and displays an outer epithelial lining only. Organoids derived from all tissue entities recapitulate key features of endometrial epithelium. Notably, EMT organoids show solitary long-ciliated cells, while euEMT and ecEMT organoids exhibit shorter, more dispersed cilia. Scale bars: 50 μm. EMT = endometrium from women without endometriosis; euEMT = eutopic endometrium from endometriosis patients; ecEMT = ectopic endometrium from endometriosis patients

All organoids recapitulated key epithelial features, such as the epithelial cell adhesion molecule (EpCAM) staining pattern, paired box protein 8 (PAX8)-positive secretory cells, and acetylated α-tubulin (ac-α-Tub)-positive ciliated cells, thereby confirming in vitro differentiation (Fig. 2). In tissue sections, EMT and euEMT displayed well-organized glandular architecture, whereas ecEMT lacked glandular morphology. Notably, EMT organoids exhibited long cilia on solitary cells (Fig. 2A), whereas euEMT and ecEMT organoids presented shorter and more dispersed cilia (Fig. 2B, C), suggesting altered ciliogenesis. Organoids demonstrated long-term expandability by sustaining 22 passages (11 months) and successful cryopreservation.

### Hormone treatment response and local estrogen biosynthesis

**Fig. 3:**
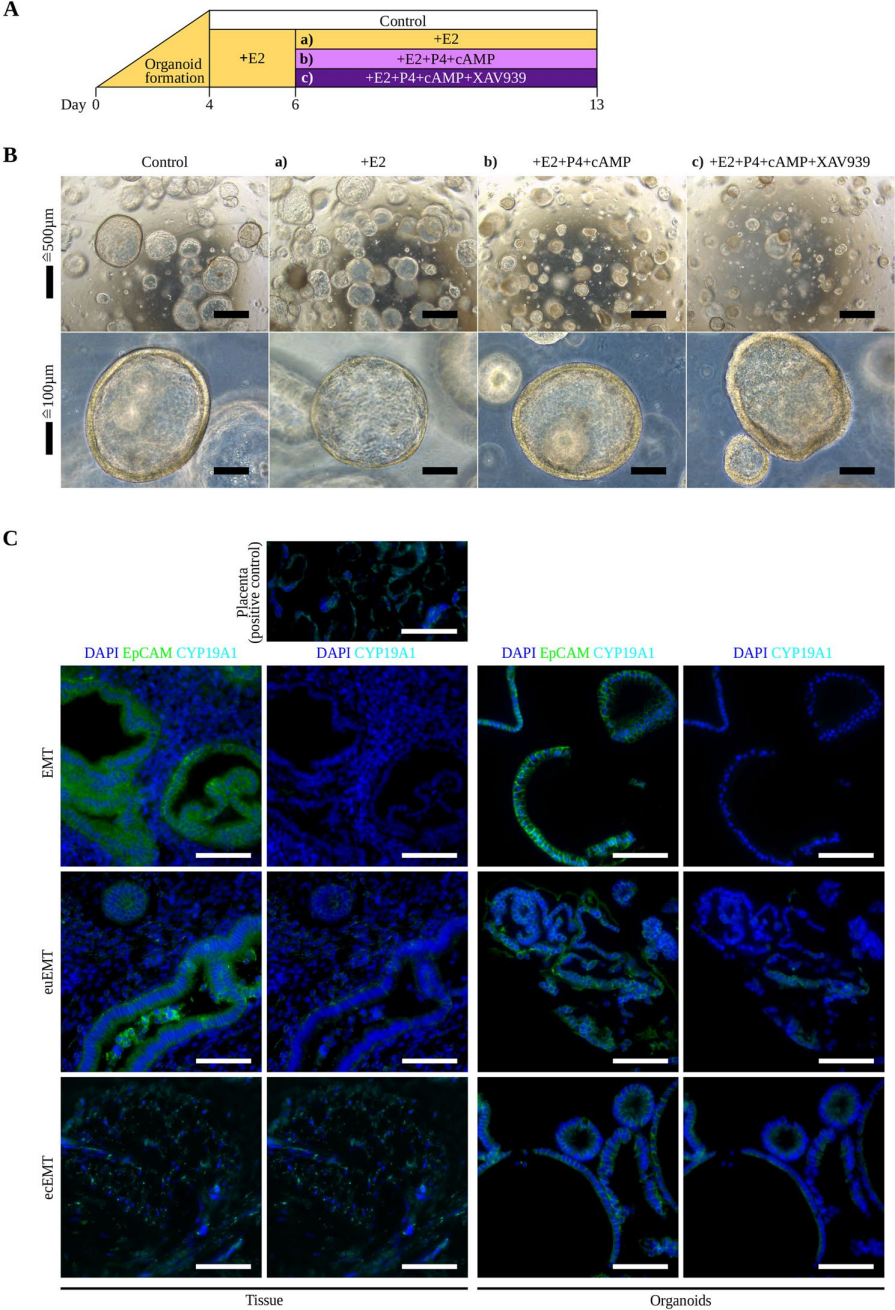
Hormonal treatment protocol, organoid morphology after hormonal treatment and analysis of *CYP19A1* expression. **A** Schematic of the hormone stimulation protocol, aligned with the full development and differentiation period of the organoid epithelium within a single passage. Organoids were seeded and allowed to form until day 4, then primed with 10 nM E2. On day 6, organoids were either left untreated (Control) or treated with **a**) 10 nM E2, **b**) 10 nM E2 + 1 µM P4 + 1 µM cAMP, or **c**) 10 nM E2 + 1 µM P4 + 1 µM cAMP + 10 µM XAV939. Organoids were harvested on day 13 for qPCR. **B** Representative brightfield images of EMT, euEMT, and ecEMT on day 13 show organoid morphology under the respective treatment conditions. **a**) E2 treatment leads to an increased number of large-sized organoids compared to Control. **b**) Addition of P4 + cAMP results in smaller, denser organoids with thickened epithelial lining, suggesting increased differentiation. **c**) Supplementation of XAV939 further enhances epithelial folding and an irregular apical surface. **C** Immunofluorescence staining of untreated EMT, euEMT, and ecEMT tissue and organoids. DAPI (blue) stains nuclei, EpCAM (green) differentiates epithelial from stromal compartments, and CYP19A1 (cyan) indicates intrinsic estrogen production. Placenta tissue serves as a positive control. No CYP19A1 expression is detected in EMT tissue or organoids. In euEMT, disseminated CYP19A1 expression appears in stroma and epithelium, with the epithelial signal recapitulated in organoids. In ecEMT, the tissue lacks glandular architecture, but scattered CYP19A1 expression is consistently seen in tissue and organoids. euEMT and ecEMT originate from the same *CYP19A1* qPCR-positive donor. Scale bars: 50 μm. E2 = 17β-estradiol; P4 = progesterone; cAMP = 8-bromadenosine-3`5`-cyclic monophosphate; XAV939 = WNT/β-catenin pathway inhibitor; CYP19A1 = aromatase

To model menstrual cycle conditions and mimic follicular and luteal phase in vitro, organoids were pretreated with 10 nM 17β-estradiol (E2), followed by exposure to 10 nM E2, additionally combined with 1 µM progesterone (P4) and 1 µM cAMP, further amended with 10 µM XAV939, or left untreated as a control (Fig. 3A). In terms of morphology, E2-treated organoids increased in number and size, whereas the morphologically observed enhanced differentiation of P4 + cAMP supplemented organoids was further strengthened by XAV939 (Fig. 3B). When the possibility of endogenous estrogen production was explored, *CYP19A1* expression was undetectable (CT > 40) in all EMT (13/13) organoids and most euEMT (9/12) and ecEMT (20/21) samples. Detectable expression (CT 33.4–35.4) was observed in euEMT and ecEMT organoids from one donor (Fig. 3C). In this case, aromatase expression was stable in untreated euEMT, variably detectable after E2, and absent after P4 + cAMP ± XAV939. These findings suggest that aromatase expression may be sporadically present in endometriosis patients. The observed absence of aromatase following P4 treatment may indicate a suppressive effect, warranting further investigation.

### E2 induces significant downregulation of *ESR1* expression

Next, we assessed the basal hormone receptor expression levels of estrogen receptor α (*ESR1*) to examine whether there are differences in expression patterns between groups before treatment. Quantified by qPCR, we observed a non-significant trend of decreased baseline *ESR1* expression in euEMT and ecEMT from endometriosis patients compared to EMT.

**Fig. 4:**
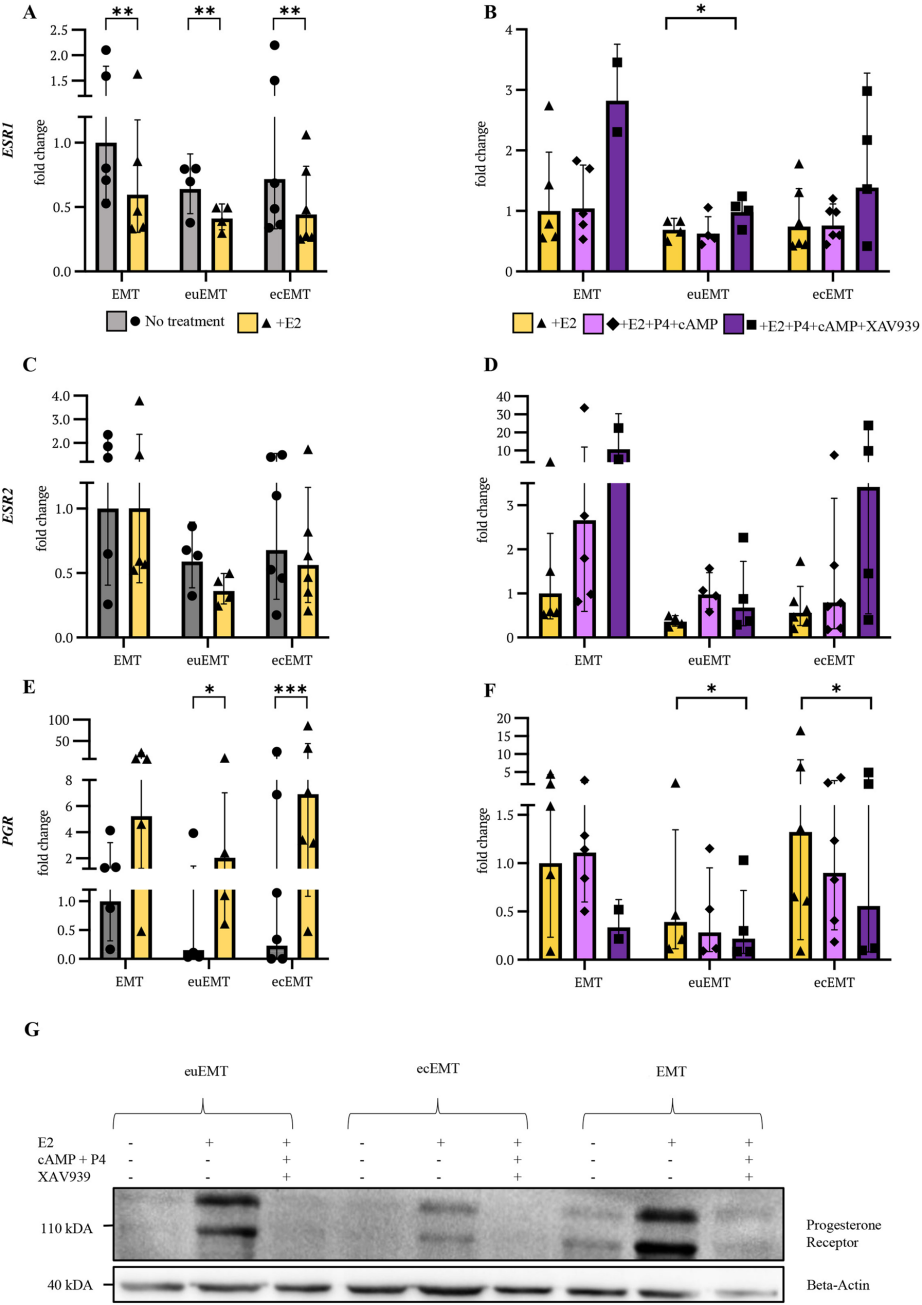
Expression of hormone receptors and response to hormonal treatment in organoids. Relative mRNA expression levels of *ESR1*, *ESR2*, and *PGR* in EMT, euEMT, and ecEMT organoids at baseline and after hormonal stimulation. Organoids were treated according to protocol (Fig. 3A) and harvested on day 13 for RT-qPCR. **A**, **C**, **E** Expression shown as fold change relative to the mean of untreated EMT organoids. **B**, **D**, **F** Expression shown as fold change relative to the mean of E2-treated EMT organoids. **A-F** Each bar represents the geometric mean of independent biological replicates, with error bars indicating the geometric standard deviation; individual data points are plotted as symbols. Statistical analysis was performed on log-transformed data. Statistical tests: **A**, **C**, **E** Two-way ANOVA, Tukey’s post hoc test; **B**, **D**, **F** mixed-effects analysis with Tukey’s post hoc test. Significance levels: *p < 0.05, **p < 0.01, ***p < 0.001. **G** Western blot analysis of the PGR levels (anti-PGR antibody recognizing both isoforms PGRA and PGRB) in EMT, euEMT, and ecEMT, after the indicated in vitro stimulation. E2 = 17β-estradiol; P4 = progesterone; cAMP = 8-bromadenosine-3`5`-cyclic monophosphate; XAV939 = WNT/β-catenin pathway inhibitor; EMT = endometrium from women without endometriosis; euEMT = eutopic endometrium from endometriosis patients; ecEMT = ectopic endometrium from endometriosis patients

Upon E2 stimulation, a highly significant decrease of *ESR1* expression in all samples was detected, in EMT by 40.3% (*p = 0.0015), euEMT by 35.7% (**p = 0.0085), and ecEMT by 38.2% (**p = 0.0012) (Fig. 4A). Furthermore, we identified donor-specific biological variability as the most pronounced (****p < 0.0001) source of variation. P4 + cAMP did not affect *ESR1* expression. However, with the addition of XAV939, we observed an increase in *ESR1* levels, particularly present in EMT (Fig. 4B). These findings suggest that WNT signaling influences the regulation of *ESR1* expression, which may be disrupted in endometriosis patients.

### Minimal *ESR2* expression across all organoid groups

The basal expression of estrogen receptor β (*ESR2*) appeared to be very low throughout all organoid groups (> 32.5 CT, compared to *ESR1* > 22.4 CT), irrespective of their tissue of origin. As expected and consistent with the low amount of *ESR2*-RNA being at the detection limit, high variance between sample triplicates and means of group entities was noticed. Although none of the following observations reached statistical significance, a consistent downward directionality in *ESR2* expression in euEMT and ecEMT was identified. The addition of E2 did not influence *ESR2* in any group (Fig. 4C). Treatment with P4 + cAMP resulted in a pattern of higher *ESR2* values, which reached consistently detectable levels (CT > 29.0) and was most pronounced in EMT (Fig. 4D). Supplementation with XAV939 further elevated *ESR2* expression in EMT and ecEMT organoids.

### Differential *PGR* dynamics in endometriosis patient-derived organoids and regulation by WNT signaling

Compared to EMT, basal progesterone receptor (*PGR*) expression levels were 4.3-fold lower in euEMT and 6.6-fold lower in ecEMT. We discovered a marked increase in *PGR* expression after E2 treatment, with a mean of 5.2-fold increase (*p* = 0.0687) in EMT, 13.5-fold (*p = 0.0155) in euEMT, and 29.9-fold (***p = 0.0007) in ecEMT (Fig. 4E). Subsequent treatment with P4 + cAMP did not lead to the expected downregulation of *PGR* expression, as would typically occur through the physiological P4-mediated feedback mechanism [38]. However, addition of XAV939 to the “secretory phase” hormonal stimulation cocktail led to *PGR* downregulation (Fig. 4F). This finding suggests that WNT inhibition is necessary for the regulation of P4 signaling in organoids.

To validate the dynamics of *PGR* levels in the organoid model, we repeated the stimulation protocol on representative lines and performed Western blotting on protein lysates (Supplement 5). As shown in Fig. 4G, the basal level of *PGR* was confirmed to be lower in endometriosis-derived organoids compared to EMT organoids. Moreover, stimulation with E2 led to a strong induction of *PGR*, adequately mimicking the “follicular phase” response. Importantly, the addition of the full “secretory phase” stimulation components (E2, P4, cAMP, and XAV939) resulted in *PGR* downregulation in all cases.

## Discussion

This study investigated the effects of hormonal stimulation and modulated WNT signaling on the expression of steroid hormone receptors across organoid groups. Our findings revealed differences in basal receptor levels, hormone responses, and microenvironmental requirements for proliferation between non-endometriotic and endometriosis-derived organoids. Remarkably, rather than resembling endometrium from women without endometriosis (EMT), the eutopic endometrium (euEMT) shares more similarities with the ectopic endometrium (ecEMT). These results propose shared epithelial compartment defects in eutopic and ectopic endometrium, thereby providing new insights into endometriosis pathophysiology and highlighting the value of patient-derived organoids as a model system.

Comparisons of culture conditions revealed a distinct WNT activation requirement: EMT and euEMT organoids required a higher WNT pathway-promoting signal by supplementation of the exogenous WNT ligand WNT3a and RSPO1, whereas ecEMT favored culture conditions with RSPO1-only medium. The introduction of XAV939 further modulated receptor responses, indicating that WNT/β-catenin signaling influences not only organoid formation and proliferation but also hormonal sensitivity.

Aromatase (*CYP19A1*) was undetectable in EMT, confirming previous reports in which aromatase was not found in the tissue of women without endometriosis [22, 23]. Sporadic, low-level aromatase was evident in a small subset of euEMT and ecEMT organoids, supporting the hypothesis of aberrant local estrogen production in some endometriotic lesions [39, 40]. However, local estrogen production cannot be excluded, as aromatase represents only one of several enzymes involved in endometrial estrogen regulation. Notably, the sporadic aromatase expression observed in some organoids diminished upon P4 treatment, aligning with its therapeutic role. However, the rare occurrence of CYP19A1 expression in our samples suggests patient-specific variability, warranting larger cohort studies.

Although the following differences did not reach statistical significance, basal *ESR1* expression appeared lower in euEMT and ecEMT compared to EMT, aligning with previous findings and suggesting altered E2 sensitivity in endometriosis-derived tissues [5, 41]. Given the role of *ESR1* in facilitating *PGR* expression, these findings may contribute to understanding mechanisms of progesterone resistance in endometriosis. Interestingly, E2-treatment led to a significant decrease in *ESR1* expression across all groups, contrasting with studies reporting *ESR1* upregulation after E2 exposure [25, 26]. However, these studies utilized a shorter E2-treatment period of 2–4 days. The 3D organoid system, which has been proven to recreate the in vivo microenvironment more realistically than conventional 2D cultures do, may reveal regulatory feedback mechanisms, such as receptor desensitization or proteasomal degradation, that restrict sustained *ESR1* expression in response to prolonged high-dose E2. These observations may indicate a necessity to adapt an extended E2 treatment period to imitate the menstrual cycle’s proliferative phase more accurately. P4 and cAMP did not affect *ESR1* expression. However, the induction of *ESR1* occurred with the inclusion of XAV939 in EMT, thereby revealing the requirement for WNT/β-catenin pathway inhibition for adequately mimicking the luteal phase in organoids. Additionally, the lack of *ESR1* increases in euEMT and ecEMT suggests a disrupted mechanism in endometriosis.

Notably, *ESR2* expression was low across all tested groups. This contradiction to previously published data could be due to the reductionist nature of the organoid model, which excludes the stromal compartment [11, 12].

The reduced *PGR* basal expression in euEMT and ecEMT supports the theory of impaired progesterone signaling in endometriosis patients. These findings, confirmed on both transcriptional and protein levels, align with the concept of progesterone resistance as a defining feature of endometriosis, which seems to be present in euEMT beforehand [20, 42]. While E2 induces *PGR*, which is consistent with the well-established role of estrogen in priming *PGR* synthesis [4], the subsequent P4-mediated downregulation occurred only with XAV939 supplementation. These results underscore the importance of WNT pathway inhibition in properly modeling the secretory phase and progesterone responsiveness in organoids.

While our study provides valuable new insights into the biology of endometriosis, several limitations must be acknowledged. Donor-specific variability and relatively small sample size must be considered when extrapolating conclusions to a large population of patients, particularly the limited number of control samples from women without endometriosis. Expanding the cohort in future experiments will be essential to increase the robustness and generalizability of these findings. Our focus on the epithelial compartment precludes an assessment of stromal influences, which are known to modulate hormonal signaling and epithelial behavior. Therefore, future studies should incorporate epithelial and stromal co-cultures to better replicate the in vivo environment and intercellular interactions. Further, differences in organoid number and size for evaluating media optimization and morphological organoid variation after drug treatment were qualitatively assessed rather than quantified, which limits the precision of comparison.

## Conclusion

Both euEMT and ecEMT organoids showed a non-significant trend toward lower *ESR1* expression relative to EMT, suggesting a possible shared attenuation in E2 sensitivity. *ESR2* levels were low across all groups but showed comparable directional changes in endometriosis patients’ organoids. *PGR* expression was likewise reduced in both endometriosis-derived organoid types, probably reflecting a mutual feature of progesterone resistance. Upon E2 treatment, *PGR* was strongly induced in euEMT and ecEMT, with a more pronounced increase than in EMT. However, despite a steep increase at the transcript level in endometriosis organoids as determined by qPCR, *PGR* protein levels did not reach those observed in the control line by Western blot analysis, potentially indicating reduced P4 signaling capacity. *PGR* downregulation by P4 required WNT pathway inhibition in all groups. In addition, aromatase expression was exclusive to euEMT and ecEMT.

Together, these convergent patterns of receptor expression, hormone responsiveness, WNT/β-catenin activation effects, and aromatase presence suggest that eutopic organoids from endometriosis patients mirror the properties of ectopic organoids more closely than those of organoids from women without endometriosis. This supports the hypothesis of endometriosis as a systemic disease and suggests an intrinsic dysregulation of eutopic endometrium with a change in homeostatic mechanisms, potentially predisposing endometrial tissue to *ex-utero* migration. Therefore, our findings provide a strong foundation for future investigations into the pathophysiology of endometriosis and the development of novel therapeutic strategies.

## Supplementary Information

Supplementary Material 1.

Supplementary Material 2.

## Data Availability

Data generated and analyzed during this study are included in this published article and its supplementary information files. Additional datasets, including original qPCR data, ΔΔCT calculations, and the detailed protocol for organoid establishment, are available from the corresponding author upon reasonable request.

## Abbreviations

ac-α-Tub: Acetylated α-tubulin
ANOVA: Analysis of variance
cAMP: 8-bromadenosine-3`5`-cyclic monophosphate
CT: Cycle threshold
CYP: 19A1 Aromatase
E2: 17β-estradiol
ecEMT: Ectopic endometrium from endometriosis patients
EMT: Endometrium from women without endometriosis
EpCAM: Epithelial cell adhesion molecule
ESR1: Estrogen receptor alpha
ESR2: Estrogen receptor beta
euEMT: Eutopic endometrium from endometriosis patients
GAPDH: Glyceraldehyde-3-phosphate dehydrogenase
P4: Progesterone
PAX8: Paired box protein 8
PGR: Progesterone receptor
RSPO1: R-Spondin 1
RT: Room temperature
RT-qPCR: Real-time quantitative polymerase chain reaction
SD: Standard deviation
WB: Western Blot
WNT: WNT/β-catenin pathway
XAV939: WNT/β-catenin pathway inhibitor
