# Supplementary material for: Eutopic echoes ectopic: organoid-based evidence of shared hormone dysregulation in endometriosis
Source: Reprod Biol Endocrinol. 2026 Apr 23;24:46. doi: 10.1186/s12958-026-01559-4 (PMC13112633; doi:10.1186/s12958-026-01559-4)
Supplement: Supplementary file 1 — Supplementary Material 1. [file 12958_2026_1559_MOESM1_ESM.docx]

Supplement Data

**Supplement 1: Materials**

Listed in order of use as described in the Methods section.

***Reagents and Chemicals***

| **Product** | **Manufacturer** | **REF / Catalogue number** |
| --- | --- | --- |
| Dulbecco's Phosphate Buffered Saline (DPBS) | Gibco | 14190-094 |
| Collagenase type I | Gibco | 17100017 |
| Rock inhibitor (Y-27632 dihydrochloride) | Bio-techne | 1254/10 |
| Advanced DMEM/F12 Reduced Serum Medium (ADF) | Gibco | 12634-010 |
| Red Blood Cell Lysis Buffer | Roche | 11814389001 |
| Cultrex Reduced Growth Factor Basement Membrane Extract, Type 2 (Cultrex) | R&D Systems | 3533-005-02 |
| TrypLE™ Express Enzyme | Gibco | 12604-013 |
| Cryo-SFM Plus freezing medium | PromoCell | C-29922 |
| 4% Paraformaldehyde (PFA) | Morphisto | 11762.01000 |
| Epredia HistoGel | Fisher Scientific | 12006679 |
| Roticlear | Roth | A538.5 |
| TRIS | Roth | AE15.2 |
| Ehylenediaminetetraacetic acid disodium salt dihydrate (EDTA) | Sigma | E5134 |
| Triton X-100 | Sigma-Aldrich | X100 |
| Blocking solution (Normal Donkey Serum) | Abcam | AB7475 |
| Antibody Dilution Reagent | DAKO | S3022 |
| DAPI (1:1000) | Thermo Scientific | 62248 |
| Mowiol 4-88 | Roth | 0713.2 |
| 17ß-Estradiol (E2) | Sigma-Aldrich | E4389 |
| Progesterone (P4) | Sigma-Aldrich | P7556 |
| 8-Bromadenosine-3`5`-cyclic monophosphate (cAMP) | Biolog | B007 |
| XAV939 | Sigma-Aldrich | X3004 |
| ß-Mercaptoethanol | Sigma-Aldrich | M3148 |
| RLT-buffer | Qiagen | 79216 |
| RNeasy Mini Kit | Qiagen | 74104 |
| Qubit RNA Broad Range Assay | Invitrogen | Q32852 |
| cDNA Synthesis Kit | Biozym | 331470X |
| TaqMan Fast Universal PCR Master Mix | Applied Biosystems | 4352042 |
| 2x Laemmli buffer | Biorad | 1610737 |
| Milk Powder, 500g | Carl Roth | 68514-61-4 |
| BSA, 100g | Sigma-Aldrich | A9647 |
| PageRuler Plus Protein prestained Ladder | Thermo Scientific | 26619 |
| Tween 20 | Sigma-Aldrich | P9416 |
| Cytiva Amersham ECL Prime Western-Blot-Detection Reagent | Thermo Scientific | RPN2236 |
| TEMED | Sigma-Aldrich | T9281 |
| APS | Biomol | 50404 |
| Acrylamid/Bis Solution | Serva | 10687.01 |
| Glycin | Merk | 1.04201.1000 |
| SDS Pellets, 500g | Roth | CN30.2 |
| NaCl | Merk | 1.06404.1000 |

***Western Blot SDS running buffer:***

10x SDS running buffer:

- Tris (Trisma Base, MG 121,14) -> 0,25 M
- Glycin (MG 75,06) -> 1,93 M
- SDS* (MG 288,38) -> 1 %

Diluted to 1x with Aqua dest

***Equipment and Consumables***

| **Product** | **Manufacturer** | **REF / Catalogue number** |
| --- | --- | --- |
| 400 µm cell strainer | PluriSelect | 43-50400-01 |
| 24-well plate | Falcon | 351147 |
| 48-well plate | Falcon | 35178 |
| 1000µl pipette tip | Sapphire | 778353 |
| 5ml conical tube | Biozym | 710440 |
| Nunc CryoTube | Thermo Scientific | 363401 |
| Freezing container (Mr.Frosty) | Thermofisher | 5100-0001 |
| Microscope slides (Superfrost Plus Adhesion) | Epredia | J1800AMNZ |
| Steamer (Vitacuisine compact) | Tefal | VS400334 |
| G26 cannula (Sterican, 0.45x25mm) | BBraun | 4657683 |
| Qubit 4 Fluorometer | Thermo Fisher | [Q33238](https://www.thermofisher.com/order/catalog/product/Q33238#/Q33238) |
| 7500-Fast Real-Time PCR System | Applied Biosystems |  |
| Trans-Blot Turbo Transfer Pack (0,2 µm PVDF) | Biorad | 1704156 |
| ChemiDoc MP Imaging System | Biorad Laboratories | 12003154 |
| Trans-Blot Turbo Transfer System | Biorad Laboratories |  |
| Mini Protean short plates | Biorad | 1653308 |
| Mini Protean spacer plates with 1 mm | Biorad | 1653311 |
| Mini Protean comb 10 wells | Biorad | 1653359 |

***Antibodies Immunofluorescence***

| Primary antibody | Secondary antibody |
| --- | --- |
| Anti EpCAM, goat, 1:200  (R&D Systems, CatNr: Af960) | Alexa Fluor Plus 488, donkey anti-goat (Invitrogen, CatNr: A32814) |
| Anti PAX8, rabbit, 1:200  (Proteintech, CatNr: 10336-1-AP) | Alexa Fluor Plus 555, donkey anti-rabbit (Thermofisher, CatNr: A32794) |
| Anti-acetylated alpha-tubulin, mouse, 1:1000 (Sigma-Aldrich, CatNr: T7451) | Cy5 Affinity Pure+A4: O16, donkey anti-mouse (Jackson Immuno Research, CatNr: 715-175-151) |

***Antibodies Western Blot***

| Primary antibody | Secondary antibody |
| --- | --- |
| Anti Progesterone Receptor 1:1000  (Cell Signaling, CatNr: 8757) | HRP goat anti-rabbit  (Thermofisher, CatNr: G-2134) |
| Anti beta Actin 1:2000  (Sigma-Aldrich, CatNr: A-5441) | HRP goat anti-mouse  (Thermofisher, CatNr: G-21040) |

***Taqman Primer***

| Gene | Assay ID | Manufacturer |
| --- | --- | --- |
| GAPDH | Hs99999905_m1 | Thermofisher, CatNr: 4331182 |
| ESR1 | Hs01046816_m1 | Thermofisher, CatNr: 4331182 |
| ESR2 | Hs01100353_m1 | Thermofisher, CatNr: 4331182 |
| PGR | Hs01556702_m1 | Thermofisher, CatNr: 4331182 |
| CYP19A1 | Hs00903411_m1 | Thermofisher, CatNr: 4331182 |

**Supplement 2: Composition of medium for human endometrial organoid forming and propagation**

| Base medium ADF++ | Company | Catalouge Number | Final Concentration |
| --- | --- | --- | --- |
| Advanced DMEM/F12 Reduced Serum Medium (ADF) | Gibco | 14190-094 | 1x |
| GlutaMAX | Thermo Scientific | 35050038 | 1% |
| HEPES (1M) | Gibco | 156630080 | 1% |

| Product | Company | Catalouge Number | Final Concentration |
| --- | --- | --- | --- |
| WNT3a | Self produced | * | 0,25 |
| R-Spondin (RSPO1) | Self produced | ** | 10% |
| EGF | Gibco | PHG0311 | 0.1 µg/ml |
| FGF-10 | Preprotech | 100-26-100UG | 0.1 µg/ml |
| Noggin | Preprotech | 120-10C-100UG | 0.1 µg/ml |
| A83-01 | Merck | 616454 | 0,5 µM |
| Y-27632 | Tocris | 1254/10 | 0,01 mM |
| N-2 | Gibco | 17502001 | 1x |
| B27 supplement | Invitrogen | 17504001 | 1x |
| N-Acetylcysteine (NAC) | Sigma | A9165-5G | 1,25 mM |
| Nicotinamide (NIC) | Sigma | N0636 | 5 mM |
| Primocin | InvivoGen | ant-pm-2 | 0,1 mg/ml |
|  |  |  | **In ADF++** |

| **Cell lines:** | **Reporter cell line:** |
| --- | --- |
| *L-Wnt+3A | 293T Wnt Reporter |
| **293T HA-Rspo1 |  |

**Supplement 3: Patient Data**

***Lines used in experiments***

| Patient | Organoid lines | Tissue origin | rASRM | #ENZIAN |
| --- | --- | --- | --- | --- |
| 1 | EMT_1 | Eutopic endometrium | No endometriosis | No endometriosis |
| 2 | EMT_2 | Eutopic endometrium | No endometriosis | No endometriosis |
| 3 | EMT_3 | Eutopic endometrium | No endometriosis | No endometriosis |
| 4 | ecEMT_4 | Peritoneal endometriotic lesion | Stage I | P1, T-/-, B1/0 |
| 5 | euEMT_5 ecEMT_5 | Eutopic endometrium Pelvic endometriotic lesion | Stage III | P3, O2/0, Tm-/2+, B1/1 |
| 6 | euEMT_6 ecEMT_6 | Eutopic endometrium Peritoneal endometriotic lesion | Stage IV | P3, O3/2, T2+/T2+, A1, B2/1, F (Appendix) |
| 7 | euEMT_7 | Eutopic endometrium | Stage II | P1, T+/-, B1/0, FB |

***Biobank***

***Cell isolation with successful organoid establishment***

| Patient | Organoid lines | Tissue origin | rASRM | #ENZIAN |
| --- | --- | --- | --- | --- |
| 1 | EMT_1 | Eutopic endometrium | No endometriosis | No endometriosis |
| 2 | EMT_2 | Eutopic endometrium | No endometriosis | No endometriosis |
| 3 | EMT_3 | Eutopic endometrium | No endometriosis | No endometriosis |
| 4 | ecEMT_4 | Peritoneal endometriotic lesion | Stage I | P1, T-/-, B1/0 |
| 5 | euEMT_5 ecEMT_5  ecEMT_5.2 | Eutopic endometrium Pelvic endometriotic lesion  Endometriotic cyst | Stage III | P3, O2/0, Tm-/2+, B1/1 |
| 6 | euEMT_6 ecEMT_6  ecEMT_6.2 | Eutopic endometrium Peritoneal endometriotic lesion  Endometriotic cyst | Stage IV | P3, O3/2, T2+/T2+, A1, B2/1, F (Appendix) |
| 7 | euEMT_7  ecEMT_7 | Eutopic endometrium  Peritoneal endometriotic lesion | Stage II | P1, T+/-, B1/0, FB |
| 8 | euEMT_8  ecEMT_8 | Eutopic endometrium Endometriotic cyst | Stage IV | P1, T1l + A2, B2l |
| 9 | ecEMT_9 | Endometriotic cyst | Stage II | P3, O2l, T2l |
| 10 | ecEMT_10 | Endometriotic cyst | Stage III | P1, O2r, T1r, B1r |
| 11 | euEMT_11  ecEMT_11 | Eutopic endometrium  Peritoneal endometriotic lesion | Stage IV | P3, O3/2, T3-/2-, A1, B2/2, FA, FO Cervix |
| 12 | ecEMT_12 | Peritoneal endometriotic lesion | Unknown | P3, A1, B1 |
| 13 | euEMT_13 | Eutopic endometrium | Unknown | P3, A3, C1, FA |
| 14 | euEMT_14 | Eutopic endometrium | - | Tubal endometriosis |
| 15 | euEMT_15 | Eutopic endometrium | Stage IV | P2 T3+/3+ A2 B3/2 C3 |
| 16 | euEMT_16 | Eutopic endometrium | Stage III | P1 O2/0 T3+/0+ B2/1 |
| 17 | euEMT_17 | Eutopic endometrium | Stage II | P2 B1/0 |
| 18 | euEMT_18 | Eutopic endometrium | Stage II | P3 T+/+ A1 B1/1 |
| 19 | euEMT_19 | Eutopic endometrium | Stage II | P2, T+/+, A2, B1/2, C2 |
| 20 | euEMT_20 | Eutopic endometrium | Stage II | P2 T1+/0+ A1 B1/0 C1 |
| 21 | ecEMT_21.2 | Endometriotic cyst | Stage III | P2, O2left, O1right, T1 left, B1right |
| 22 | euEMT_22  ecEMT_22  ecEMT_22.2 | Eutopic endometrium Peritoneal endometriotic lesion  Endometriotic cyst | Stage III | P2, T+/+, A2, B1/2, C2, Fa, FU left |
| 23 | ecEMT_23.2 | Endometriotic cyst | Stage IV | P2, O2, T2 (-/-), B1, FA. |
| 24 | euEMT_24  ecEMT_24 | Eutopic endometrium  Endometriotic cyst | Stage IV | P3, O3, O1, T1, B1, FA |
| 25 | euEMT_25  ecEMT_25 | Eutopic endometrium  Peritoneal endometriotic lesion | Unknown | P2, O1 left, T-/+, FA |
| 26 | euEMT_26 | Eutopic endometrium | Stage II | P3 T+/- B1/0 |
| 27 | euEMT_27 | Eutopic endometrium | Stage II | P3, T1/1(+), B1 |
| 32 | euEMT_32 | Eutopic endometrium | Stage I | P1 |
| 33 | EMT_33 | Eutopic endometrium | No endometriosis | No endometriosis |

**Total number of EMT organoid lines: 4**

**Total number of euEMT organoid lines: 19**

**Total number of ecEMT organoid lines: 17**

***Cell isolation with unsuccessful organoid establishment***

| Patient | Organoid lines | Tissue origin | rASRM | #ENZIAN |
| --- | --- | --- | --- | --- |
| 8 | ecEMT_8.2 | Peritoneal endometriotic lesion | Stage IV | P1, T1l + A2, B2l |
| 9 | ecEMT_9.2 | Pelvic endometriotic lesion | Stage II | P3, O2l, T2l |
| 16 | ecEMT_16 | Peritoneal endometriotic lesion | Stage III | P1 O2/0 T3+/0+ B2/1 |
| 17 | ecEMT_17 | Peritoneal endometriotic lesion | Stage II | P2 B1/0 |
| 18 | ecEMT_18 | Pelvic endometriotic lesion | Stage II | P3 T+/+ A1 B1/1 |
| 19 | ecEMT_19 | Deep infiltrating endometriotic lesion | Stage II | P2, T+/+, A2, B1/2, C2 |
| 20 | ecEMT_20 | Peritoneal endometriotic lesion | Stage II | P2 T1+/0+ A1 B1/0 C1 |
| 21 | ecEMT_21 | Peritoneal endometriotic lesion | Stage III | P2, O2left, O1right, T1 left, B1right |
| 23 | ecEMT_23 | Peritoneal endometriotic lesion | Stage IV | P2, O2, T2 (-/-), B1, FA. |
| 26 | ecEMT_26 | Peritoneal endometriotic lesion | Stage II | P3 T+/- B1/0 |
| 27 | ecEMT_27 | Peritoneal endometriotic lesion | Stage II | P3, T1/1(+), B1 |
| 28 | ecEMT_28 | Peritoneal endometriotic lesion | Stage IV | P1, T1l + A2, B2l |
| 29 | ecEMT_29 | Pelvic endometriotic lesion | Stage II | P1, B2/0 |
| 30 | ecEMT_30 | Pelvic endometriotic lesion | Stage III | P1 O0/1 T3+/3+ A2 |
| 31 | ecEMT_31 | Peritoneal endometriotic lesion | Stage IV | P3 O1/0 T3(m)/3+ A3 B3/3 C1 |

**Total number of cell lines, in which organoid establishment was unsuccessful: 15**

**Supplement 5: Western Blot raw data**


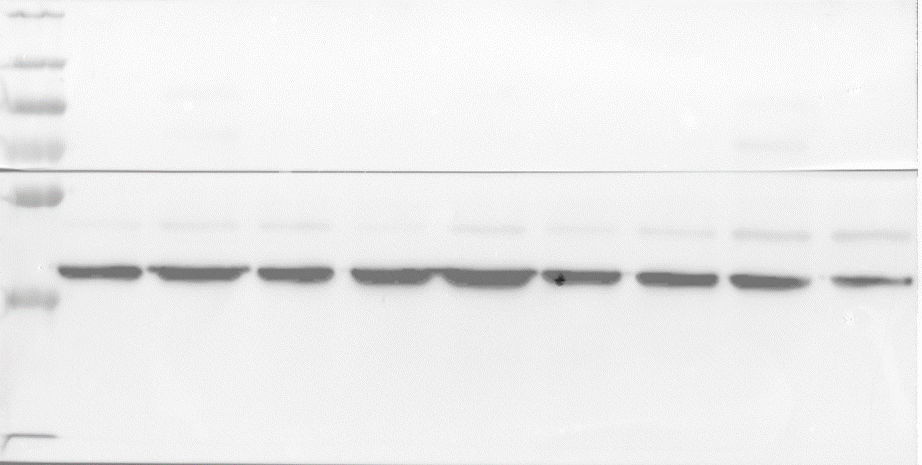


Exposure time: 0 min 18 s


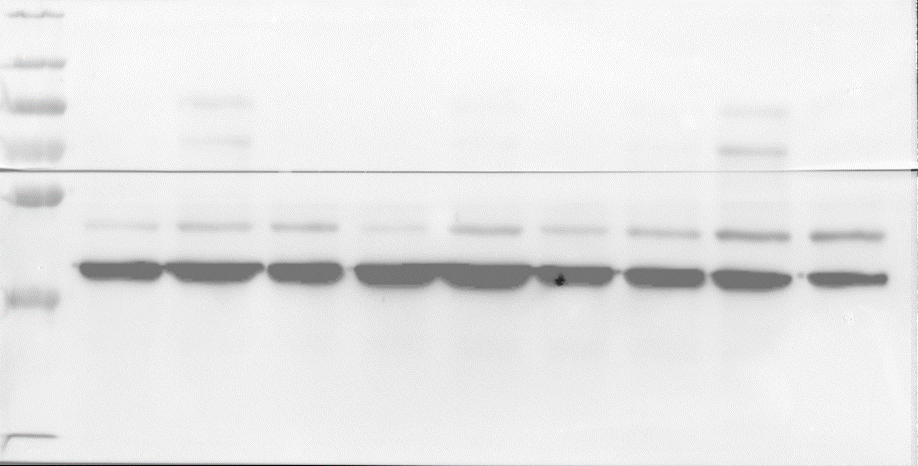


Exposure time: 0 min 41 s


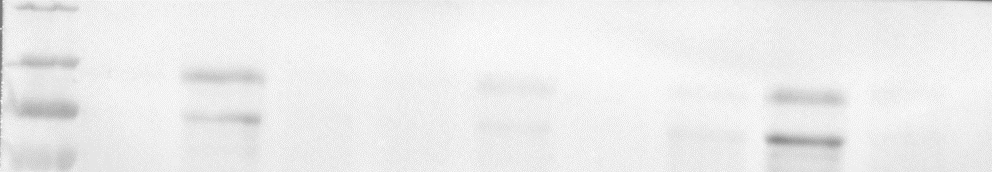


Exposure time: 3 min 12 s


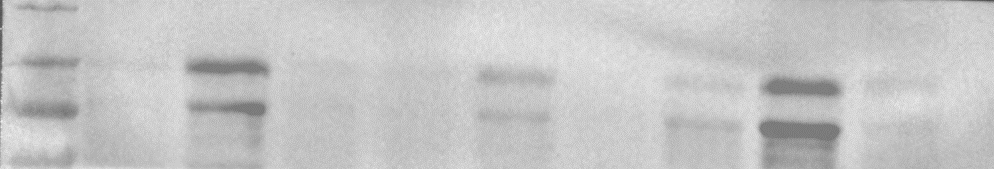


Exposure Time: 3 min 44 s
